# PLGA particulate delivery systems for subunit vaccines: Linking particle properties to immunogenicity

**DOI:** 10.1080/21645515.2015.1117714

**Published:** 2016-01-11

**Authors:** A. L. Silva, P. C. Soema, B. Slütter, F. Ossendorp, W. Jiskoot

**Affiliations:** a Division of Drug Delivery Technology, Leiden Academic Center for Drug Research, Leiden University, Leiden, The Netherlands; b Intravacc (Institute for Translational Vaccinology), Bilthoven, The Netherlands; c Cluster BioTherapeutics, Leiden Academic Center for Drug Research, Leiden University, Leiden, The Netherlands; d Department of Immunohematology and Blood Transfusion, Leiden University Medical Center, Leiden, The Netherlands

**Keywords:** antigen, adjuvant, dendritic cells, delivery systems, microparticles, nanoparticles, PLGA, subunit, vaccine

## Abstract

Among the emerging subunit vaccines are recombinant protein- and synthetic peptide-based vaccine formulations. However, proteins and peptides have a low intrinsic immunogenicity. A common strategy to overcome this is to co-deliver (an) antigen(s) with (an) immune modulator(s) by co-encapsulating them in a particulate delivery system, such as poly(lactic-co-glycolic acid) (PLGA) particles. Particulate PLGA formulations offer many advantages for antigen delivery as they are biocompatible and biodegradable; can protect the antigens from degradation and clearance; allow for co-encapsulation of antigens and immune modulators; can be targeted to antigen presenting cells; and their particulate nature can increase uptake and cross-presentation by mimicking the size and shape of an invading pathogen. In this review we discuss the pros and cons of using PLGA particulate formulations for subunit vaccine delivery and provide an overview of formulation parameters that influence their adjuvanticity and the ensuing immune response.

## Introduction

Vaccination consists of the administration of antigens in order to elicit an adaptive antigen-specific immune response and confer long-term protection against subsequent exposure to the antigen.^1^ Traditional vaccine formulations, consisting of either live attenuated or killed pathogens, have been very successful in the last century to drastically reduce the incidence of widespread infectious diseases.^2,3^ Still, despite their success,^4,5^ this traditional vaccine approach has not resulted in effective vaccines against disease like AIDS, tuberculosis, or cancer. These issues have led to the demand for alternatives and vaccine development shifted from using whole inactivated pathogens to subunits of the pathogen. These subunits may be natural or recombinant antigenic proteins, peptides, capsular polysaccharides or any specific part of the pathogen which has been demonstrated to stimulate a protective immune response. Examples of subunit vaccines include hepatitis B, tetanus, diphtheria, pneumococcus and human papillomavirus (HPV) vaccines. However, the need for eliciting both humoral and cellular immune responses has limited the efficacy of subunit vaccines. While subunits are safer than whole pathogens, they generally are less immunogenic, demanding the use of adjuvants.^5^ Adjuvants are immunostimulatory molecules and/or delivery systems ^6^ used in vaccine formulations to enhance the magnitude of antigen-specific immune responses.

Immunostimulatory molecules activate the immune system through their interaction with specific receptors of APCs, which recognize evolutionary conserved molecular motifs associated with groups of pathogens, the pathogen-associated molecular patterns (PAMPs). These membrane-bound pattern recognition receptors (PRRs) include nucleotide-binding oligomerization domain (NOD)-like receptors (NLRs), C-type lectin receptors (CLRs) and Toll-like receptors (TLRs). PAMPs have been shown to enhance and modulate the immune response when mixed, conjugated, or co-delivered together with antigen.^7,8^ This knowledge opens the door to the rational design of vaccine formulations that co-deliver PAMPs to increase the immunogenicity of the antigen.

Next to immunostimulatory molecules, subunit vaccines may benefit from encapsulation in particulate delivery systems, which include microparticles (MP) (> 1 μm) and nanoparticles (NP) (< 1000 nm). Particles may promote immunogenicity through the following mechanisms:

1. Stability improvement of the antigen: particulate delivery systems can protect encapsulated or associated antigen from chemical and enzymatic degradation and rapid clearance via the kidneys, resulting in increased residence time ^1,6^
2. Controlled antigen release: particulate formulations can be tailored to serve as extra- and/or intracellular depot for sustained release of the antigen, increasing antigen exposure to DCs and prolonged antigen presentation, respectively ^9^
3. Facilitated DC uptake: particulate delivery systems can mimic the size and shape of an invading pathogen, which facilitates uptake by DCs ^7,10^
4. Targeted delivery: particles *per se* are passively directed to APCs because of their particulate form, but can also be specifically targeted to specific tissues or subsets of immune cells (like DCs) via targeting moieties, such as TLR ligands or DC-specific antibodies ^11-14^
5. Enhanced cross-presentation: particles may facilitate endosomal escape, which is a known mechanism leading to antigen cross-presentation by DCs and induction of a CTL response ^15,16^
6. Concomitant delivery of multiple components: particulate formulations can co-deliver a combination of molecules, such as (multiple) antigens and/or immunostimulatory molecules and/or targeting ligands, mimicking pathogens and facilitating uptake by APCs and stimulating immune activation ^9,10^
7. Regulation of the type of immune response: immunological properties of particles can be tailored by changing their size, surface charge, or hydrophobicity ^1,6^

Owing to the potential synergistic effect of all the above-mentioned effects, particles can also serve to decrease the dose of antigen required to elicit an immune response.^7^

A large number of particulate systems has been reported, such as polymeric particles, liposomes, virus-like particles, virosomes, immunostimulating complexes (ISCOMs), emulsions, and inorganic nanobeads. Among these, poly(D,L-lactide-*co*-glycolide) (PLGA)-based delivery systems have been particularly well studied and are promising candidates for antigen delivery.^17^ Since the initial description of PLGA particle as potential adjuvants by O'Hagan *et al*,^18^ PLGA particles have been formulated in a wide variety of ways resulting in various size, charge, antigen stability, loading capacity and release profiles. These key formulation aspects can greatly affect the potency of the vaccine and will be discussed in detail in this review.

## PLGA particulate systems for subunit vaccine delivery

PLGA and its derivatives are aliphatic polyesters that are available in different ratios of lactic acid and glycolic acid, various molecular weights, and type of end groups (ester-terminated (capped) or carboxylic acid terminated (uncapped)). PLGA polymers have been widely studied over the past few decades for several biomedical applications because of their excellent safety records, varying from sutures to bone reconstruction, as well as in implants and particles for sustained drug delivery, and it has long been approved for parenteral human use by the FDA.^19-21^ After their administration, PLGA particles undergo degradation by bulk erosion, during which water diffuses into the polymeric matrix, hydrolyzing the ester bonds throughout the polymer and reducing its molecular weight until degradation products are formed that can be dissolved.^6^ This process increases porosity of the matrix, allowing the sustained release of the entrapped material as degradation continues. Finally, PLGA is hydrolyzed into the original monomers, lactic acid and glycolic acid, which are by-products of various metabolic pathways and are not associated with significant toxicity.^22^ The degradation rate of PLGA is related to molecular weight, hydrophilicity and crystallinity, but also other factors such as pH of the medium, water uptake rate, process of ester hydrolysis, swelling ratio and degradation by-products.^6,23^ Lower molecular weight molecules degrade faster, as shorter molecules can be more easily hydrolyzed and dissolved, leaving the polymeric matrix. Higher hydrophilicity can also lead to faster degradation: the hydophilicity is mainly influenced by the monomers' ratio, with glycolic acid being more hydrophilic than lactic acid, so the higher the content of glycolic acid, the more hydrophilic, increasing hydrolysis rate.^22^ An exception to this rule is the co-polymer with 50:50 lactide:glycolide ratio, which has the fastest degradation rate, even among polymer compositions with higher glycolic acid content. This is due to the influence of crystallinity: the higher the crystallinity, the slower the degradation, and at a 50:50 ratio the polymer is the least crystalline, resulting in the fastest degradation rate.^6,24^ Uptake of PLGA particles by APCs may further expedite the degradation of PLGA, as the acidic environment of the endosomal compartment (pH ˜4.5 – 6.5)^25^ accelerates degradation compared to physiological pH (pH 7.4) since low pH catalyzes breakage of the ester linkage of the polymer backbone.^26,27^ Thus, depending on the type of PLGA polymer used, PLGA particles can be made with distinct release kinetics.^12,28-30^ Next to release characteristics various other physical traits of PLGA particles can be manipulated including particle size, size distribution, zeta potential, polydispersity index, encapsulation efficiency and drug loading.^23^ PLGA particles can be prepared by a variety of different methods, most commonly used for protein and peptide antigens being the double emulsion with solvent evaporation method.^22^ Using this method, all previously mentioned characteristics can be controlled during the assembly of the particles and can be produced according to good manufacturing practice in a scalable, affordable and reproducible way.^22^ Several analytical methods can be used to characterize the physicochemical properties of particles and encapsulated antigens.^31,32^ (see Table 1 for examples of commonly used techniques).

**Table 1.** Examples of analytical methods for characterization of antigen-containing PLGA particles.

Particle characteristic	Method
Particle size	Dynamic light scattering Nanoparticle tracking analysis Light obscuration Scanning electron microscopy Transmission electron microscopy Atomic force microscopy
Density	Density gradient centrifugation Helium compression pycnometry Resonant mass measurement
Crystallinity	X-ray diffraction Differential scanning calorimetry
Surface chemistry	X-ray photoelectron spectroscopy Nuclear magnetic resonance spectroscopy
Surface charge	Electrophoresis Laser Doppler velocimetry
Surface hydrophobicity	Hydrophobic interaction chromatography Contact angle measurement Two-phase partitioning
Antigen content, release and integrity	Bicinchoninic acid assay SDS-PAGE High performance size-exclusion chromatography Reverse-phase high performance liquid chromatography Enzyme-linked immunosorbent assay Fluorescence spectroscopy UV/VIS spectroscopy Fourier transform infrared spectroscopy Mass spectrometry

While many properties are favorable and controllable, there are also drawbacks in using PLGA particles as a delivery system, especially concerning the stability of encapsulated protein antigens, which will be discussed in more detail later on. Therefore, antigen stability after encapsulation and storage should be evaluated, and each formulation should be specifically customized for each antigen, accordingly to its properties.^5^ Still, considering that naked antigen has a very short residence time because of rapid degradation and clearance upon administration,^1,6^ the drawbacks are neglectable compared to the advantage of protection from the surrounding environment offered by encapsulation.

## 1 PLGA particle characteristics affecting adjuvanticity

Depending on the preparation method and conditions, PLGA particles can be made with diameters ranging from about 80 nm to 250 μm.^8^ Moreover, various experimental conditions can be chosen and varied, such as type of solvent and polymer, polymer molecular weight, polymer concentration, type and concentration of surfactants, homogenization mechanism, duration and intensity, or volume ratio of phases. Each of these different factors can affect the particle size, size distribution, zeta potential, encapsulation efficiency, drug loading and release profile,^23^ which in turn affect the immunogenicity of the formulation. In the following sections we will systemically review these effects.

### Particle size

Particle size of PLGA particles is one of the most critical factors affecting their interaction with APCs as well as their biodistribution. Particle size is strongly dependent on the preparation process parameters, such as type and concentration of surfactants, polymer concentration, phase volume ratios and homogenization speed.^23^ Higher polymer concentration leads to bigger particles, due to higher viscosity of the oil phase, making it harder to break the droplets. Higher inner water-in-oil emulsion (w1/o) to outer aqueous phase (w2) ratios [(w1/o)/w2] also lead to larger particles, due to higher solidification rate, while higher surfactant concentrations lead to more stable emulsions and can produce smaller particles.^23^ The method of homogenization and its speed are also among the most important factors: for instance, microparticles are usually produced by using homogenizers and/or magnetic stirring, whereas nanoparticles are produced by sonication, since the higher the homogenization speed, the smaller the particles.

Particle size is known to influence the loading capacity, depot formation and release kinetics.^33-35^ The particle size and size distribution affect the antigen release rate, as the total surface area for protein delivery depends on the particle size.^23^ On the one hand, the smaller the particle, the faster the antigen release, as smaller particles have a larger surface area, and therefore a greater proportion of antigen located near their surface, which can lead to a higher burst release.^36,37^ On the other hand, microparticles have larger cores from which the encapsulated antigen slowly diffuses out, and require more time to be degraded, resulting in lower release rates.^37^

Smaller particles are generally regarded as more effective delivery vehicles, since their size would allow easier travel through epithelia and other biological barriers and efficiently reach target tissues.^38-40^ The impact of antigen delivery system size on the resultant immune response also depends on the route of administration employed. Particles in the size range of 20-50 nm are suitable for transport through lymphatic vessels to reach lymph nodes, where they can increase the probability of immune cell interaction, but are not suitable for inhalable vaccination.^1,6^ In contrast, large particles (500–2000 nm) require cellular transport by APCs to be delivered to lymph nodes.^39^ However, there is still no definitive answer to which size PLGA particles are the most effective for vaccine delivery, and results of different studies comparing nanoparticles and microparticles are somewhat contradictory.^29,34,35^ A strong correlation between particle size and the mechanism of antigen uptake, processing and presentation by APCs has been reported in different studies.^33-35,41-43^ APCs are known to take up and process particles with dimensions comparable to viruses and bacteria.^44^ The way APCs take up the vaccine can determine how they process the antigen. Soluble antigens are preferentially presented by the MHC class II pathway and are poorly cross-presented. Particles in the range of 20-200 nm are efficiently taken up by DCs via endocytosis or pinocytosis and facilitate the induction of cellular immune responses, whereas microparticles of 0.5–5 µm are taken up via phagocytosis or macropinocytosis, mainly generating humoral responses.^34,35,45^ Particles larger than 10 µm are hardly taken up, leading to defective immune activation.^46-48^ It has also been postulated that large microparticles (> 10 µm) preferentially attach to the surface of macrophages, thus serving as an extracellular depot system for continuous antigen release.^35^ Comparative studies about the effect of PLGA particle size on the observed immune response have been summarized in Table 2. These studies suggest that the efficiency of internalization significantly affects the resulting immune response. However, one should bear in mind that particle properties other than size may also affect their fate and biological effects (see following sections).

**Table 2.** Comparative studies about the effect of PLGA particle size on the observed immune response.

Formulation	Particle size	Antigen/TLRL	*In vitro / in vivo*	Adminstration route	Response	References
PLGA MPs	5 μm, 12 μm	HBsAg protein	*In vitro and in vivo*	Pulmonary	5 µm > 12 µm MPs uptaken by rat alveolar macrophages; Ab responses: 5 µm > 12 µm MPs	^49^
PLGA NPs & MPs	200 nm, 500 nm, 1 μm	BSA protein	*In vitro and in vivo*	s.c.	Ab responses: 200 nm ˜500 nm < 1 μm particles.	^42^
PLA NPs & MPs	200-600 nm, 2-8 µm	HBsAg protein	*In vitro and in vivo*	i.m.	NPs >> MPs uptaken by macrophages; MPs ↑ anti-HBsAg Ab responses and ↑ IL-4 secretion related to a Th2 response; NPs ↑ IFN-γ production and ↑ Ab isotype related to a Th1 response.	^50^
PLA MPs	< 2 μm, 2-8 μm, 10-70 μm, 50-150 μm	TT	*In vivo*	i.m.	Ab responses ↑ by 2-8 µm MPs > > 10-70 μm ˜50-150 μm.	^48^
PLGA NPs & MPs	500-600 nm, 3.5 μm	TT	*In vivo*	i.m.	NPs and MPs mixed together ↑ Ab responses > NPs ˜ MPs alone	^51^
PLGA NPs & MPs	17 μm, 7 μm, 1 μm, 300 nm	OVA / Cpg ODN	*In vitro and in vivo*	i.p.	Particle uptake and upregulation of MHC class I and CD86 expression and ↑ OVA-specific CD8^+^ T cells and ↑ IgG2a:IgG1 following the same size trend: : 17 μm << 7 μm < 1 μm < 300 nm	^34^
PLGA NPs & MPs	300 nm,> 20 μm	OVA / poly(I:C)	*In vitro and in vivo*	s.c.	NPs >> MPs internalized by DCs and ↑ CD8^+^ T cell activation *in vitro*; vaccination with NPs ↑ OVA-specific CD8^+^ T cells & Ab production, MPs did not	^47^
PLGA NPs & MPs	600 nm, 1 – 1.5 μm	OVA	*In vitro*	n/a	MPs > NPs induced *in vitro* MHC class I Ag cross-presentation	^52^

Ab: antibody; Ag: antigen; <: less/lower than; >: more/higher than; <<: much less/lower than; >>: much more/higher than; ˜: similar; ↑: increased/high: ↓: decreased/low

The size of MPs should not be too large, as Thomas *et al.* showed that hepatitis B surface antigen (HBsAg) in PLGA MPs with a size of 5 µm elicited a significantly higher serum antibody response than 12 µm MPs upon pulmonary administration in rats, while confocal imaging showed that smaller particles were taken up more efficiently by alveolar macrophages.^49^ A study investigating the immunogenicity of differently sized PLGA particles (200, 500 and 1 µm) encapsulating bovine serum albumin (BSA) showed that 1 µm-sized particles were capable of inducing stronger IgG responses *in vivo* than 200 and 500 nm NPs following immunization via intranasal, oral and s.c. routes in mice.^42^

Similar studies were conducted also with PLA MPs encapsulating HBsAg, showing that MPs of 2-8 µm induced stronger anti-HBsAg antibody responses than NPs of 200-600 nm after intramuscular (i.m.) immunization of rats.^50^ However, PLA NPs were efficiently taken up by macrophages, whereas PLA MPs primarily were found attached to the surface of the macrophages. Immunization with PLA MPs promoted IL-4 secretion, upregulated MHC class II molecules and favored a Th2 response, whereas immunization with PLA NPs was associated with higher levels of IFN-γ production, upregulation of MHC class I molecules along with antibody isotypes related to a Th1 response.^50^ Comparable results were obtained with i.m. vaccination of rats with tetanus toxoid (TT) in PLA particles.^48^ So, the choice of particle size may be dependent on the type of immune response desired: NPs tend to favor a Th1 bias, whereas MPs promote Th2 based responses.

After comparing the immunogenicity of TT loaded PLGA NPs (500-600 nm) and MPs (4 µm), both types of particles were mixed together into one formulation.^51^ After i.m. immunization of rats, this mixture elicited higher antibody responses compared to the NPs or MPs alone, which elicited similar responses. A mixture of both size classes could also be considered to stimulate both Th1 and Th2 type responses.

Joshi *et al.* compared 17 μm, 7 μm, 1 μm, and 300 nm sized PLGA particles co-encapsulating ovalbumin (OVA) and CpG, by selectively recovering these particles with different centrifugation cycles. They showed a size-dependent burst release over 48 h followed by a plateau, with total OVA and CpG release ranging from 100% for 300 nm NPs to circa 10% for 17 μm MPs.^34^ In a head-to-head comparison, they observed that the efficiency of particle uptake and upregulation of MHC class I and CD86 expression on murine bone marrow-derived dendritic cells (BMDC) correlated with smaller particle size.^34^ The same trend was observed following intraperitoneal vaccination, with the 300 nm NP generating the highest antigen-specific cytotoxic T cell responses, and the highest IgG2a:IgG1 ratio of OVA-specific antibodies, in proportion to DC uptake. These results concur with our own observations, since we have recently compared PLGA NP circa 300 nm with MP > 20 μm, co-encapsulating OVA and poly(I:C), with similar compositions and release properties, for their capacity to induce MHC class I cross-presentation *in vitro* and improve immune responses *in vivo*.^47^ NPs were efficiently internalized by DCs *in vitro*, whereas MP were not. Subcutaneous vaccination of C57BL/6 mice with NPs resulted in significantly better priming of Ag-specific CD8^+^ T cells compared to MP. NP also induced a balanced T_H_1/T_H_2-type antibody response, whereas MP failed to increase antibody titers.^47^ These studies suggest that particulate vaccines should be formulated in the nano-size range to achieve efficient uptake, MHC class I cross-presentation and CTL responses.

### Controlled antigen/adjuvant release

In addition to their ability to protect antigens, favor antigen uptake by APCs and enhance the immune response, controlled release systems can extend antigen release for prolonged periods of time.^53,54^ Antigen/adjuvant release from PLGA particles is dependent on a variety of factors, such as size, polymer composition, porosity of the matrix, antigen loading or the way it is associated with PLGA particles, i.e. encapsulated/entrapped or adsorbed on the surface. In the first case, antigen release depends on the degradation, erosion or dissolution of the polymer; whereas in the second case it is dependent on the interactions between the polymer and the antigen.^55^ Entrapment of the antigen within the particle matrix protects antigen from external environment but may lead to incomplete release, which could lead to a weak immune response; in contrast, adsorption may lead to high burst release, prematurely releasing the antigen from the particulate carrier before uptake by DCs, which can lead to deficient immune responses.^36^ Frequently, a combination of adsorbed and encapsulated antigen occurs, resulting in a characteristic triphasic release profile with an initial burst followed by a lag phase and a final sustained release phase of the encapsulated antigen dictated by polymer erosion.^55,56^ Initial burst release of antigen can be generally explained by 2 mechanisms: either by the release of antigens that are adsorbed or located in the surface layer, or by antigen escape through pores and cracks that may form during the fabrication process.^57-59^ Several factors affect burst release: higher hydrophilicity, lower molecular weight and lower polymer concentration can lead to higher burst release.^23,30,60^ By adding salts to the inner water phase (w1), the porosity of the resulting particles can be controlled by increasing the osmotic gradient and the flux of water from w2 into the w1/polymer phase, increasing antigen release rate.^47^ Suspensions of sugars^61^ or salts in the oil phase are expected to act in a similar way, resulting in a major increase in water uptake, e.g., by incorporation of suspended NaCl, which has been shown with PLGA films.^62^ A larger inner surface, induced by a higher porosity of the particles, can potentially increase the uptake of the release medium into the particles and accelerate the drug pore-diffusion and release.^63^ After burst, the release of encapsulated material from such systems is dependent on diffusivity through the polymer barrier (a more hydrophobic polymer will create a higher barrier), porosity, size of antigen molecule and distribution throughout the matrix, leading to prolonged antigen release, thereby enhancing the duration of antigen exposure to APCs and thereby the potency of the resultant response.^64^

Antigen release kinetics regulate the antigen's exposure to the immune system. If most of the cargo is burst released immediately after immunization and before uptake, antigen will be delivered to APCs in soluble form, losing the benefit of particulate delivery.^36^ In contrast, if the release profile is too slow or incomplete, there will not be enough antigen available for presentation by APCs. For instance, Hailemichael *et al.* showed that Montanide-based persisting vaccine depots can induce specific T cell sequestration, dysfunction and deletion at vaccination sites, whereas short-lived formulations may overcome these limitations and result in greater therapeutic efficacy of peptide-based cancer vaccines.^65^ Still, sustained release of antigen/adjuvant seems crucial to properly activate DCs, whereas a low burst eliminates potential antigen loss before uptake, increasing antigen presentation and CD8^+^ T cell activation.^9,36^ Kanchan *et al.* reported that slow and continuous release of antigen/adjuvant may prolong MHC antigen presentation, which play a key role in T cell stimulation and activation, and in eliciting memory antibody responses.^66^ It has been reported that extended antigen release may enhance not only the level, but also the quality of immune responses.^35^ Johansen *et al.* demonstrated that antigenic delivery increasing exponentially over time induced more potent CD8^+^ T cell responses and antiviral immunity than a single dose or multiple equivalent doses (zero order).^33^ Shen *et al.* showed that OVA-loaded PLGA MPs enhanced exogenous antigen MHC class I cross-presentation at 1000-fold lower concentration than soluble antigen, and served as an intracellular antigen reservoir, leading to sustained MHC class I presentation of OVA for 72 h.^16^ Likewise, Waeckerle-Men *et al.* showed that MHC classes I and II-restricted presentation of proteins and peptides encapsulated in PLGA MPs (0.5 – 5 µm) was markedly prolonged and presented 50-fold more efficiently on class I molecules than soluble antigens.^67^ A difference in performance between PLGA NPs connected to the kinetics of antigen delivery was shown by Demento *et al.*, with “slow” releasing NPs eliciting prolonged antibody titers comparing to “fast” releasing ones.^9^ Moreover, “slow” release favored long-term effector-memory cellular responses. Finally, Zhang *et al.* formulated OVA-loaded PLGA NPs by encapsulating antigen within NPs or by simply mixing soluble antigen with the NPs, observing that the combined formulations induced more powerful antigen-specific immune responses than each single-component formulation. The enhanced immune responses elicited by the combined vaccine formulation may be ascribed to the combination of a depot effect at the injecton site, adequate initial antigen exposure and long-term antigen persistence leading to prolonged antigen presentation.^68^

#### Surface characteristics

Surface characteristics such as shape, hydrophobicity, and zeta potential are reported to influence phagocytic uptake by APCs. Because cells are negatively charged, cationic particles induce phagocytic uptake more efficiently than anionic particles, owing to electrostatic attraction to the negatively charged APC membranes.^69,70^ Strategies aimed at improving the efficacy of PLGA particles as antigen delivery vehicles involve coating them with ionic surfactants or polymers such as poly(ethylene glycol) (PEG), sodium dodecyl sulfate (SDS), aminodextran, chitosan, poly(ethylene imine) (PEI), poly(L-lysine), protamine or cetyltrimethylammonium bromide (CTAB).^55,71,72^ Coating can be achieved either by incorporating these agents in the particle matrix (together with the polymer or in the external aqueous phase during the emulsification process), or by adsorption to the surface of pre-formed particles by resuspending them in a solution containing the coating and incubating for a determined amount of time. Besides changing surface charge, some of these molecules have bioadhesive properties, such as chitosan,^1^ which has been employed to develop formulations for mucosal delivery. Polycations can also aid in phagosomal/endosomal escape after being internalized by APCs,^1^ potentially improving MHC class I presentation and CTL responses.

Wishke *et al.* studied the impact of the surface properties of MPs (5 – 10 μm) on phagocytosis, using BSA bearing fluorescein isothiocyanate groups (FITC-BSA) as model antigen.^72^ Modification with chitosan and DEAE-dextran resulted in stable MPs and increased cellular uptake by DCs. Positively charged PLGA MPs (1 – 5 μm) containing hepatitis B surface antigen (HBsAg) were prepared with cationic agents stearylamine and PEI in the external aqueous phase.^69^ Compared to unmodified formulations, positive surface charge enhanced both the systemic and mucosal immune response upon immunization of rats via the intranasal route. PLGA MPs containing recombinant HBsAg and coated with chitosan were developed for nasal immunization.^73^ The modified PLGA microspheres showed the lowest nasal clearance rate and a 30-fold increase of serum IgG levels. OVA-loaded PLGA NPs coated with N-trimethyl chitosan (TMC) were more efficiently taken up by DCs and showed a longer nasal residence time than uncoated particles.^74^

Protamine, a cationic polypeptide, has been used as a surface-coating material because of its ability of increasing cell penetration.^75^ Protamine coating of PLGA MPs (˜7 μm) encapsulating the purified phospholipase A2 (PLA2) from bee venom or OVA injected s.c. in mice led to increased antibody and T-cell responses as compared to uncoated particles (˜3 μm), most likely mediated by an increased uptake. In another study from the same group, combination of adsorbed protamine and CpG (˜8 μm) resulted in strong PLA2-specific antibody responses and the induction of the Th1-associated isotype IgG2a.^76^ However, when the MHC class I-restricted OVA peptide SIINFEKL was encapsulated into bare PLGA MPs, protamine- or chitosan-coated MPs with CpG either covalently coupled or physically adsorbed on their surface,^77^ only the uncoated MPs with adsorbed CpG mediated a prominent CTL response in mice after s.c. immunization, with failure of the other formulations being ascribed to the low release of antigen and CpG.

In conclusion, modifying the surface charge may help increase particle uptake efficiency and result in a stronger immune response, especially when considering mucosal delivery. Furthermore, modification of the particle surface using either polycations or polyanions has been used to create cationic or anionic particles to which charged antigens/adjuvants can be adsorbed, which may be beneficial to improve antigen stability.

#### Targeted delivery to DCs

##### TLRL co-delivery in PLGA systems

One of the greatest benefits of particulate antigen delivery systems is their ability to co-deliver antigens and immunostimulatory molecules simultaneously to the same APCs.^78^ The concomitant delivery of TLRLs and antigens in PLGA particles has been proven successful to enhance antigen-specific CTL responses.^77,79^ The appropriate selection of the TLRL for co-delivery will determine the bias toward Th1 or Th2 responses.^78^ Furthermore, as most pathogens simultaneously present multiple TLR agonists to APCs, the combination of multiple TLRLs may result in a synergistic effect and a promising strategy to induce strong protective immune responses.^8^ Over the last decades, some of these ligands have been used in several vaccine formulations to target and activate TLRs.

Most commonly delivered TLRLs in PLGA particulate systems include CpG, a ligand to TLR9 which is known to induce a MHC class I driven antigen presentation;^80-83^ poly(I:C), a TLR3L analog to viral double-stranded RNA, which is also known to enhance cross-priming of CD8^+^ cytotoxic T lymphocytes;^79,84-86^ monophosphoryl lipid A (MPLA), a detoxified form of lipid A derived from LPS which is a potent TLR4 agonist;^10,87-94^ the TLR1/2 agonist Pam3CSK4, a synthetic tripalmitoylated lipopeptide that mimics the acylated N-terminus of bacterial lipoproteins;^14,95,96^ and small synthetic molecules like single-stranded RNA analogs and imidazoquinolines, such as resiquimod (R848),^11^ recognized by TLR7 and TLR8. Co-delivery of TLRLs and antigen with PLGA particles consistently increased the effectiveness of the adjuvants, with the importance of co-encapsulation being shown in several studies.^10,79,81^ A combination of TLR agonists can act synergistically to increase MHC class I-restricted presentation of exogenous antigen, resulting in more potent cellular responses.^11,14,86^ A summary of PLGA vaccine formulations containing TLRLs can be found in Table 3.

**Table 3.** Examples of reports of PLGA formulations using Toll-like receptor ligands and their immunological effects.

Receptor	Ligand	Formulation	Antigen	In vitro / in vivo	Adminstration route	Response	References
TLR 1/2	Pam3CSK4	PLGA NPs (˜350 nm)	OVA24 peptide	*In vitro and in vivo*	s.c.	TLR 2 stimulation ↑ MHC class I presentation of OVA24-NPs by DCs *in vitro* and ↑ prolonged Ag presentation and CD8^+^ T cell activation *in vivo* after adoptive transfer of NP-loaded DCs	^96^
	Pam3CSK4	PLGA NPs (˜500) and MPs (˜2 μm; μm)	CS_252-260_ coupled to Pam3CSK4 (Pam-CS_252-260_)	*In vivo*	i.p	Pam-CS_252-260_ particles ↑ cytolytic activity > CS_252-260_-MPs or sPam-CS_252-260_; 500 nm NPs > 2 μm ˜ μm MPs inducing CTL responses	^95^
TLR 3	Poly(I:C)	(DEAE)–dextran- PLGA MPs (˜3 μm)	FITC-BSA	*In vitro*	n/a	poly(I:C) coated-MPs ↑ expression of CD80, CD86, and CD83 at the DC surface ˜ cytokine cocktail or ↑ concentrations of sPoly(I:C).	^84^
TLR 4	MPLA	PLGA MPs (1 – 10 μm)	OVA_323-39_ peptide; MUC1 mucin peptide	*In vivo*	s.c.	Ag/MPLA-MPs ↑ T cell proliferative response and production of IFN-γ by T cells, eliciting a specific Th1 immune response > Ag-MPs or Ag mixed with alum	^87,88^
	MPLA	PLGA NPs (350 – 450 nm)	OVA protein	*In vitro and in vivo*	i.p. or s.c.	OVA/MPLA-NPs ↑ CD8^+^ T cell proliferative responses & IFN-γ *in vitro and* >13-folds increase in clonal expanded CD4^+^ T cells *in vivo* > OVA-NPs	^89^
	MPLA	PLGA NPs (˜300 nm)	HBcAg protein	*In vivo*	s.c.	HBcAg/MPLA-NPs ↑ Th1 cellular response with predominant IFN-γ profile > sHBcAg, sHBcAg/sMPLA, or HBcAg-NPs	^91^
	MPLA	PLGA NPs (˜500 nm)	HBcAg_129–140_	*In vivo*	s.c.	HBcAg_129–140_/MPLA-NPs ↑ Th1-type response > control formulation of HBcAg_129–140_in CFA	^92^
	MPLA	PLGA NPs (350 – 450 nm)	OVA; MUC1 lipopeptide (BLP25)	*In vitro and in vivo*	n/a	OVA/MPLA-NPs ↑ *in vitro* and *in vivo* antigen-specific primary Th1 immune responses > OVA-NPs or sOVA/sMPLA after adoptive transfer of antigen-pulsed DCs; MUC1/MPLA-NPs delivery to DCs ↑ MUC1 reactive T cells *in vitro* > MUC1-NPs, MPLA-NPs, sMUC1, or sMUC1 with MPLA-NPs	^10^
	7-acyl lipid A	PLGA NPs (350 – 410 nm)	TRP2_180-188_peptide	*In vivo*	s.c.	TRP2_180-188_/7-acyl lipid A_-_NPs ↑ CD8^+^ T cell-mediated anti-tumor immunity and therapeutic anti-tumor effect and levels of IFN-γ and pro-inflammatory Th1-related cytokines > TRP2_180-188_-NPs	^90^
	MPLA	PLGA NPs (˜80 nm)	TRP2_180-188_peptide	*In vitro and in vivo*	i.d.	NP ↑ uptake *in vitro* and *in vivo*; TRP2_180-188_/MPLA-NPs ↓ growth of s.c. inoculated B16 melanoma cells in a prophylactic setting > TRP2_180-188_-NPs, sTRP2_180-188_/sMPLA	^93^
	MPLA or RC529	PLGA MPs (3 – 5 μm)	gp120 protein; MenB	*In vivo*	i.p.	Ag adsorbed on TLRL-MPs ↑ IgG serum titers > Ag adsorbed-MPs with sTLRL.	^94^
TLR 9	CpG ODN	PLGA NPs (˜300 nm)	Tetanus toxoid (TT)	*In vitro and in vivo*	s.c.	TT/CpG-NPs ↑ antigen-specific T cell proliferation *ex vivo* & IFN-γ secretion and 16-fold IgG titers > sTT/sCPG; co-encapsulation ↑ Th1 and Th2 immune responses toward Th1 type bias.	^80^
	CpG ODN	PLGA MPs (μm)	OVA protein; CpG-OVA conjugate	*In vitro and in vivo*	s.c.	OVA/CpG-MPs were uptaken by DCs *in vitro*; OVA/CpG-MPs ↑ Ag-specific CD4^+^ and CD8^+^ T cells ˜ CPG-OVA conjugates *in vivo*. In a tumor challenge, MPs caused complete tumor regression in 4 out of 5 mice.	^82^
	CpG ODN	PLGA MPs (μm)	PLA2 protein	*In vivo*	s.c.	PLA2/CPG-MPs ↑ PLA2-specific Ab responses and ↑ Th1-associated isotype IgG2a. The effect of CpG ↑ when protamine was co-encapsulated for complexation of CpG.	^76^
	CpG ODN	bare, chitosan-coated, and protamine-coated PLGA MPs (μm)	SIINFEKL peptide	*In vivo*	s.c.	Only uncoated SIINFEKL-MPs with adsorbed CpG ↑ IFN-γ secreting and SIINFEKL-specific CD8^+^ T cells.	^77^
	CpG ODN	PLGA MPs (˜1 – 1.5 μm) coated with CTAB or DSS	p55 gag or gp120 env proteins	*In vitro and in vivo*	i.m.	CpG adsorbed to PLGA-CTAB MPs co-administered with gp120 env or p55 gag proteins adsorbed to PLGA-DSS MPs ↑ Ag-specific serum IgG titers, as well as CTL responses against p55 gag > sCp/sAg,	^102^
	CpG ODN-chitosan complexes	PLGA 502 and 752 MPs (˜1 – 2 μm)	OVA protein	*In vivo*	i.d.	OVA/CpG-MPs ↑ Ab response and isotype shifting to Th1 > OVA- MPs.	^81^
TLR 9 & TLR 3	CpG ODN or Poly(I:C)	PLGA MPs (μm)	OVA protein	*In vivo*	s.c.	CpG/OVA- or poly(I:C)/OVA-MPs ↑ (i) SIINFEKL/H-2Kb tetramer positive CTLs, (ii) IFN-γ production, (iii) *in vivo* cytotoxicity and (iv) protection from vaccinia virus > to OVA-MPs with sTLRL or OVA-MPs with TLRL-MPs.	^79^
	CpG ODN & Poly(I:C)	PLGA MPs (˜0.5 - 5 μm)	OVA protein	*In vivo*	s.c.	OVA/CpG-MPs with MP-poly(I:C) ≥ IFA in eradication of preexisting tumors and suppression of lung metastases	^85^
	CpG ODN or/and Poly(I:C)	PLGA NPs (˜1 μm)	OVA protein	*In vitro*		poly(I:C)/OVA- or CpG/OVA-NPs ↑ prolonged MHC class I- & II-restricted presentation and ↑ OVA-specific CD8^+^ and CD4^+^ T cells; combination of both TLRLs synergistically ↑ MHC class I-restricted, but not class II, Ag presentation.	^86^

Ab: antibody; Ag: antigen; <: less/lower than; >: more/higher than; <<: much less/lower than; >>: much more/higher than; ≥: equal or higher than; ˜: similar; ↑: increased/high: ↓: decreased/low; CFA: complete Freund's adjuvant; sX: soluble X

Conjugation of antigens to adjuvants to increase their immunogenicity has been successfully achieved.^82,83,97-100^ This approach, however, requires processes that have to be developed and optimized for each individual antigen-adjuvant combination, whereas particulate formulations offer a more generic approach.

The best way to deliver adjuvants with PLGA particles, by either entrapment or adsorption, is yet to be resolved. The better choice likely depends on the cellular location of their target receptors: if they act on the cell surface, it might be desirable to have the adjuvant readily available on uptake; but if they need to be internalized to interact with endosomal receptors, encapsulation within the particle might be preferable.^101^

##### Targeted delivery to other DC receptors

Aside from TLR ligands, there are other targeting ligands that have been used with PLGA particles to increase the immunogenicity of subunit vaccines (see Table 4). This can be achieved by modifying the particle surface with ligands that can target specific surface receptors of APCs, by either physical association or conjugation reactions.^1,5^ Physical association is driven by electrostatic and hydrophobic interactions, whereas preformed PLGA nanoparticles with carboxyl end groups can be chemically conjugated with molecules with terminal amine groups via amide coupling reactions using carbodiimide reagents.^103^ To that end, the surface of PLGA is first derivatized by PEG-NH_2_ with functional end groups that can react with different ligands, such as biotin-PEG-NH_2_.^103^ As avidin and its homologues show very high affinity to biotin, biotinylated PEG-PLGA particles allow noncovalent binding with avidin-ligand conjugates or vice versa, allowing targeting ligands such as antibodies to be attached to PLGA particles.^103^ Interaction between PLGA particles functionalized with specific ligands and/or antibodies against DC receptors may improve targeting to DCs, increase particle uptake by DCs through receptor-mediated endocytosis and modulate DC maturation, and thereby enhance the effectiveness of the vaccine formulation.^104^

**Table 4.** Examples of studies of PLGA particles targeted to DCs.

Receptor	Formulation	Antigen / adjuvant	In vitro /in vivo	Administration route	Response compared to untargeted particles	References
Integrin, lectin and mannose receptors	PLGA MPs (˜2.5 μm) c.c. to RGD peptide; WGA; mannose-PEG_3_-NH_2_	–	*In vitro*	n/a	↑ uptake of targeted MPs	^108^
Integrin receptor	PLGA NPs (˜200 nm) c.c. to RGD peptide	OVA	*In vitro and and in vivo*	Oral	↑uptake by M cells and ↑ IgG responses *in vivo*	^107^
	PLGA MPs (˜1 μm) containing alginate or c.c. RGD-alginate	SPf66; S3	*In vivo*	i.d.	↑ Ab and cellular responses and more balanced Th1/Th2 responses; ↑ IFN-γ secretion and splenocyte proliferation	^109^
Mannose receptor	Mannan c.c. to PLGA NPs (˜400 nm)	OVA	*In vitro and and in vivo*	s.c.	↑ antigen-specific CD4^+^ and CD8^+^ T cell responses *in vitro and* and *vivo*	^113^
	Mannan-coated on or c.c. to PLGA NPs (˜400-500 nm)	–	*In vitro*	n/a	↑ DC uptake and cell surface markers (CD40, CD86) and secretion of inflammatory cytokines (IL-12, IL-6 and TNF-α)	^111,114^
DC-SIGN	PLGA MPs (2 μm) and NPs (200 nm) c.c. to humanized hD1 anti-DC-SIGN antibody	BSA; TT	*In vitro*	n/a	MPs were taken up nonspecifically; NPs effectively targeted DCs: ↑ uptake & Ag-specific T cell responses at 10–100 fold lower concentrations	^12^
DEC-205	PLGA NPs (˜200 nm) c.c. to bfFp containing anti-DEC-205 antibody fragment	OVA	*In vitro and and in vivo*	s.c.	2-fold ↑ receptor-mediated uptake of bfFp functionalized NPs *in vitro*; ↑ OVA-specific IgG responses *in vivo*	^117^
DEC-205	PLGA NPs (˜200-250) c.c. to anti-DEC-205 mAb	OVA / KRN	*In vitro and and in vivo*	Footpads	↑ antigen-specific humoral & CTL responses and promoted potent antitumor responses	^119^
DEC-205; CD40; CD11	PLGA NPs (200 nm) c.c. either with anti-DEC-205, -αCD40 or -CD11 mAbs	OVA / poly(I:C) & R848	*In vitro and and in vivo*	s.c.	↑ uptake of targeted NPs & IL-12 production and expression of IFN-γ *in vitro*; ↑ OVA-specific CD8^+^ T cell responses *in vivo*	^11^
CD40	PLGA NPs (200 nm) c.c. with anti-αCD40 mAb	OVA; HPV-E7 / poly(I:C) & Pam3CSK4	*In vitro and and in vivo*		↑ selective delivery to DCs and ↑ CD8^+^ T cell priming *in vitro*; ↑ tumor control and prolonged survival of tumor-bearing mice *in vivo*	^14^

Ab: antibody; Ag: antigen; <: less/lower than; >: more/higher than; <<: much less/lower than; >>: much more/higher than; ≥: equal or higher than; ˜: similar; ↑: increased/high: ↓: decreased/low; CFA: complete Freund's adjuvant; sX: soluble X; c.c.: chemically conjugated; bfFp: bifunctional fusion protein of strepatividin

M-cell targeting can be considered if the vaccine is administered at a mucosal tissue.^105,106^ Integrins are heterodimeric transmembrane subunits that have specific affinities toward peptides with an arginine-glycine-aspartate (RGD) sequence^103^ and are highly expressed on M-cells. Grafting of integrin-binding RGD peptides can be used to promote the uptake of NPs via interaction with β1 integrins on M-cells.^107-109^

C-type lectin receptors (CLRs) are endocytic receptors that recognize exogenous and endogenous carbohydrates which are present on the surface of DCs and macrophages.^103^ Antigens associated with specific sugar residues can target to these receptors on DCs, including the mannose receptor, DEC-205 (also known as CD205), and DC-specific intracellular adhesion molecule-3 (ICAM3)-grabbing non-integrin (DC-SIGN).^110^ Two main strategies can be used to target CLRs, either by grafting particles with specific sugar residues which are natural ligands for these endocytic receptors (e.g., sugars with terminal mannose, fucose or N-acetylglucosamine) or by coupling mAbs against them.^111,112^ Many CLRs expressed by DCs are directly implicated in immunoregulatory processes, such as antigen uptake, intracellular trafficking and antigen presentation.^110^ PLGA particles decorated with mannan, a natural polymannose isolated from the cell wall of *Saccharomyces cerevisiae*, have been designed for targeted DC delivery via mannose receptors.^111,113-116^ DEC-205 has successfully been used to target DCs *in vivo*.^112,117,118^ A study by Cruz *et al.* using antigen-loaded NPs conjugated to anti-DC-SIGN targeting antibody improved activation of antigen-specific T-cell responses at 10–100 fold lower concentrations of antigen compared to the non-targeted NPs.^12^ Similar studies targeting DEC-205, CD40 or CD11 increased uptake by DCs and CD8^+^ T cell activation, showing that targeting to specific DC receptors is a viable approach to increase the efficacy of particulate vaccines.^11,14^

## Conclusions

Vaccination with subunit antigens is not always successful due to their limited bioavailability and poor immunogenicity. Moreover, soluble antigens are often inefficiently cross-presented. Delivery systems can be used in order to overcome these problems, by protecting antigens from degradation and increase their biodistribution and ability to reach and be uptaken by APCs. The main advantages and disadvantages of PLGA-based particulate vaccine delivery systems are summarized in Table 5.

**Table 5.** Summary of the main advantages and disadvantages of PLGA-based particulate vaccine delivery systems.

Advantages	Disadvantages
• PLGA polymers are biodegradable, widely available and approved by regulatory agencies such as FDA • PLGA particles for delivery of several different agents are on the market • PLGA particles can be administered via various routes • PLGA particles may decrease toxicity of vaccine components • Particle size, surface and/or release characteristics can be tailored • PLGA particles allow controlled Ag release • PLGA particles protect Ag from degradation and elimination • PLGA particles enhance Ag uptake by APCs by mimicking size and shape of pathogens • PLGA particles enhance and prolong Ag cross-presentation efficiency • PLGA particles allow concomitant delivery of multiple vaccine components • Large surface area and surface functional groups allow conjugating of targeting moieties • PLGA particles may lead to Ag dose sparing	• Negative charge of PLGA particles is disadvantageous for particle uptake • PLGA particle preparation process must be tailored to the properties of the Ag • PLGA particles cannot be sterile filtered • Ag degradation may occur during preparation, storage and release • Ag release is often incomplete • Particle aggregation may occur • Particle size may limit crossing of biological barriers

Depending on their physicochemical characteristics, delivery systems can modulate the immune response, mainly due to direct influence in the following mechanisms: facilitated uptake by APCs, regulation of the internalization pathways and ability to endosomal escape, and interaction with specific receptors that mediate the immune response toward humoral or cellular bias. The main immunogenic properties of viruses that elicit potent immune responses may serve as a base for rational vaccine design.^120^

Most studies are clear: size plays a crucial role in vaccine efficacy. Smaller particles tend to be more immunogenic due to their easier uptake by DCs and more efficient transport in the lymphatic system, where they can reach immature DC subsets; still, microparticles can form stable antigen depots and are more suitable for inhalable pulmonary vaccination.^1^ Recent studies have suggested that smaller particles mostly induce cellular immunity while larger particles tend to induce humoral responses.^1,35^ Other important factors include release kinetics; surface characteristics; concomitant delivery of antigen and immunostimulants, allowing DCs to associate danger signals with the antigen, while co-encapsulation of multiple TLRLs may result in a synergistic effect; coating or coupling of DC-specific targeting moieties, increasing DC uptake and enhancing antigen presentation to T cells. Future developments in vaccine delivery will likely involve the combination of immunostimulants with delivery vehicles modified with DC-specific targeting ligands or antibodies.

In summary, vaccines that mimic the size, charge, release kinetics and PAMPs of pathogens may be the future of peptide-based immunotherapy of cancer and/or other diseases that cannot be treated by conventional vaccines.

## Abbreviations

Ab: Antibody
Ag: Antigen
APC: Antigen-presenting cell
BMDC: Bone marrow-derived dendritic cells
BSA: Bovine serum albumin
CD4^+^: T cell T helper cell
CD8^+^: T cell Cytotoxic T lymphocyte
CFA: Complete Freund's adjuvant
CLR: C-type lectin receptor
CpG ODN: Unmethylated cytosine-phosphodiester-guanine oligodeoxynucleotide motif
CTAB: Cetyltrimethylammonium
CTL: Cytotoxic T lymphocyte
CTLA-4: Cytotoxic T lymphocyte-associated antigen 4
DC: Dendritic cell
DEAE: Diethylaminoethyl
DNA: DNA
DOTAP: Dioleoyl-trimethylammonium-propane
DSS: Dioctylsulfosuccinate
FDA: Food and Drug Administration
FITC: Fluorescein isothiocyanate
gp: Glycoprotein
HBcAg: Hepatitis B core antigen
HBsAg: Hepatitis B surface antigen
HPLC: High-performance liquid chromatography
HPV: Human papillomavirus
i.d.: Intradermal
i.m.: Intramuscular
i.n.: Intranasal
i.p.: Intraperitoneal
IFA: Incomplete Freund's adjuvant
IgG: Immunoglobulin G
IgG1: Immunoglobulin G subtype 1
IgG2a/b: Immunoglobulin G subtype 2a/b
IL: Interleukin
INF-γ: Interferon gamma
ISCOM: Immune stimulatory complex
LPS: Lipopolysaccharide
mAb: Monoclonal antibody
M-cell: Microfold cell
Men B: Neisseria meningitidis serotype B
MHC I/II: Major histocompatibility complex class I/II
MP: Microparticle
MPLA: Monophosphoryl lipid A
NOD: Nucleotide-binding oligomerization domain receptor
NP: Nanoparticle
o/w: Oil-in-water (emulsion)
OVA: Ovalbumin
OVA17: 17-residue synthetic long peptide of ovalbumin (ISQAVHAAHAEINEAGR)
OVA24: 24-residue synthetic long peptide of ovalbumin (DEVSGLEQLESIINFEKLAAAAAK)
Pam3CSK4: Synthetic triacylated lipopeptide
PAMP: Pathogen associated molecular pattern
PEG: Poly(ethylene glycol)
PEI: Poly(ethylene imine)
PLA: Poly(lactic acid)
PLA2: Phospholipase A2
PLGA: Poly(lactic-co-glycolic acid)
Poly(I:C): Polyinosinic:polycytidylic acid
PRR: Pattern recognition receptor
RGD: Arginine-glycine-aspartate
RNA: Ribonucleic acid
RP-HPLC: Reversed-phase high-pressure liquid chromatography
s.c: Subcutaneous
SDS: Sodium dodecyl sulfate
SDS-PAGE: Sodium dodecyl sulfate polyacrylamide gel electrophoresis
SEM: Scanning electron microscopy
Th: T helper
Th1: Type 1 helper T
Th2: Type 2 helper T
TLR: Toll-like receptor
TLRL: Toll-like receptor ligand
TMC: N-trimethyl chitosan
TNF: Tumor necrosis factor
TRP1/2: Tyrosinase-related protein 1/2
TT: Tetanus toxoid
w/o/w: Water-in-oil-in-water (emulsion)
